# Diabetes prevention interventions for women after gestational diabetes mellitus: an overview of reviews

**DOI:** 10.1002/edm2.230

**Published:** 2021-02-01

**Authors:** Anne‐Mette Hedeager Momsen, Diana Høtoft, Lisbeth Ørtenblad, Finn Friis Lauszus, Rubab Hassan Agha Krogh, Vibeke Lynggaard, Jens Juel Christiansen, Helle Terkildsen Maindal, Claus Vinther Nielsen

**Affiliations:** ^1^ Section for Clinical Social Medicine and Rehabilitation Gødstrup Hospital Herning Denmark; ^2^ DEFACTUM ‐ Social & Health Services and Labour Market Corporate Quality, Central Denmark Region Aarhus Denmark; ^3^ Department of Public Health Aarhus University Aarhus Denmark; ^4^ Department of Gynaecology and Obstetrics Gødstrup Hospital Gødstrup Denmark; ^5^ Steno Partner Collaboration between Gødstrup Hospital and Steno Diabetes Center Aarhus Herning Denmark; ^6^ Cardiovascular Research Unit Department of Cardiology Gødstrup Hospital Herning Denmark; ^7^ Department of Medicine Gødstrup Hospital Herning Denmark; ^8^ Steno Diabetes Center Copenhagen Health Promotion Research Gentofte Denmark

**Keywords:** exercise/physical activity, gestational diabetes, healthcare delivery, nutrition and diet, prevention of diabetes

## Abstract

**Aims:**

To present an overview of reviews of interventions for the prevention of diabetes in women after gestational diabetes mellitus (GDM) with the overall aim of gaining information in order to establish local interventions.

**Methods:**

Six databases were searched for quantitative, qualitative or mixed‐methods systematic reviews. All types of interventions or screening programmes were eligible. The outcomes were effectiveness of reducing diabetes incidence, encouraging healthy behavioural changes and enhancing women's perceptions of their increased risks of developing type 2 diabetes following GDM.

**Results:**

Eighteen reviews were included: three on screening programmes and seven on participation and risk perceptions. Interventions promoting physical activity, healthy diet, breastfeeding and antidiabetic medicine reported significantly decreased incidence of postpartum diabetes, up to 34% reduction after any breastfeeding compared to none. Effects were larger if the intervention began early after birth and lasted longer. Participation in screening rose up to 40% with face‐to‐face recruitment in a GDM healthcare setting. Interventions were mainly based in healthcare settings and involved up to nine health professions, councillors and peer educators, mostly dieticians. Women reported a lack of postpartum care and demonstrated a low knowledge of risk factors for developing type 2 diabetes. Typical barriers to participation were lack of awareness of increased risk and low levels of support from family.

**Conclusions:**

Lifestyle interventions or pharmacological treatment postpartum was effective in decreasing diabetes incidence following GDM. Women's knowledge of the risk of diabetes and importance of physical activity was insufficient. Early face‐to‐face recruitment increased participation in screening. Programmes aimed at women following a diagnosis of GDM ought to provide professional and social support, promote screening, breastfeeding, knowledge of risk factors, be long‐lasting and offered early after birth, preferably by face‐to‐face recruitment.

## Graphical abstract

One in two women with gestational diabetes develops diabetes within 10 years. This overview explored effectiveness and determinants for participation in programmes. Conclusions: Preventive programmes should inform women about risk factors and include physical activity, healthy diet and promotion of breastfeeding. Early recruitment is important to rely on motivational factors.

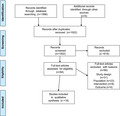

**Already known regarding gestational diabetes**

- The incidence is increasing and follow‐up is inadequate.
- One in two women with gestational diabetes develop diabetes within 10 years after birth the highest risk being within the first 5 years.

**Findings regarding women with gestational diabetes**

- Programmes including physical activity healthy diet and promotion of breastfeeding were effective in preventing diabetes.
- Recruitment should start early as this appears to be the time when women may be most motivated to make lifestyle changes.
- Emphasis should be placed on supporting women to adopt healthy lifestyles and breastfeed.
- Women lack knowledge about the risk of diabetes for themselves and their children and need professional follow‐up and social support after giving birth.

**Implications for clinical practice in women with gestational diabetes**

- Preventive programmes should be offered early in the postpartum period preferably by face‐to‐face recruitment in local healthcare settings

## 1 INTRODUCTION

Prevalence of gestational diabetes mellitus (GDM) is increasing globally, reportedly 2%–26% depending on ethnicity and the diagnostic criteria used.^1^, ^2^, ^3^ GDM is related to several adverse outcomes during pregnancy and birth.^4^ Complications include pre‐eclampsia, shoulder dystocia, children born large for gestational age, neonatal hypoglycaemia and hyperbilirubinaemia. After GDM, lifetime risk of type 2 diabetes is increased,^5^ and up to 50% of women with GDM will develop diabetes within 10 years.^6^ The highest incidence is reported within 5 years after a GDM pregnancy^7^ and varies according to the time of the postpartum examination^7^ and diagnostic criteria.^8^ A recent meta‐analysis including more than 1.3 million individuals found the risk appears to be almost 10‐fold higher for Type 2 diabetes and thereby for all‐cause mortality.^9^ GDM is also a predictor of obesity and diabetes later in life in the offspring.^10^ New data confirm that women who develop GDM suffer from a latent metabolic disorder that comes to clinical attention during pregnancy.^11^ Thus, GDM helps identify women who have a long‐standing, high‐risk cardiometabolic profile.^11^ Known postpartum risk factors are 2‐fold greater risk for elevated body mass index and >3‐fold greater risk for an abnormal oral glucose tolerance test.^9^

The worldwide increase in Type 2 diabetes has directed attention towards systematic follow‐up programmes and clinical routines established to prevent progression of GDM to manifest Type 2 diabetes.^6^ In Denmark, general practitioners are responsible for the postpartum follow‐up. However, systematic follow‐up programmes are lacking in routine clinical settings.^6^

Some current approaches are considered not to be cost‐effective,^1^, ^12^, ^13^, ^14^ although they do help in delaying or preventing diabetes in women with GDM if a structured approach is used.^15^ Adherence to preventive programmes seems challenged by women's low perception of the high risk of developing diabetes after GDM.^16^ Women with previous GDM called for better continuation of postpartum care,^17^ a finding which stresses the importance of programmes containing strategies for healthy lifestyle promotion.^18^

A systematic overview of reviews from 2017 concluded that there was’no robust evidence to support the hypothesis that non‐pharmacological interventions are effective at lowering the risk’,^17^ whereas another review concludes that any intervention is superior to no intervention.^19^ Seemingly, there is no robust consensus on the content and effectiveness of interventions or the value of screening.^20^

The present study is an overview of reviews of interventions for preventing Type 2 diabetes in women following GDM to explore the effectiveness, organization and stakeholders involved, and the perceived risks and barriers for participation in order to establish preventive local interventions.

## 2 METHODS

To perform the overview, the principles from the Joanna Briggs Institute (JBI) methodology were followed.^21^, ^22^ The protocol was registered a priori in the international prospective register of systematic reviews (PROSPERO), registration number: CRD42019131001.

### 2.1 Searching

An initial search was conducted in the Cochrane Library, the JBI Database of Systematic Reviews and Implementation Reports, PubMed, Epistemonikos and PROSPERO. This displayed numerous systematic reviews about the topic; however, only one overview of systematic reviews included randomized controlled trials (RCTs) only.^17^ Thus, an overview was decided upon that included qualitative as well as quantitative systematic reviews to draw on a broader range of evidence.

For this study, six databases (Cochrane Library, PubMed, JBI, Embase, CINAHL, Web of Science) were searched for eligible reviews following a 3‐step search strategy. An initial search of PubMed was undertaken followed by an analysis of keywords and index terms. Secondly, the search strategy developed for PubMed was refined with assistance from a research librarian for use in the other databases. Thirdly, the reference lists of all included reviews were searched to find additional reviews.

The search was limited to reviews, systematic reviews, meta‐analysis and meta‐synthesis published in English, Danish, Norwegian and Swedish, published from 2009 to 2019. Predefined search filters regarding’systematic reviews’ were applied or specific keywords were included in the search story in the databases, which have no predefined filters (Appendix 1: Search history).

### 2.2 Inclusion

Eligible for inclusion was peer‐reviewed quantitative, qualitative or mixed‐methods systematic reviews including meta‐analysis or meta‐synthesis reporting on the effect on incidence of diabetes among women following GDM, organizational aspects and stakeholders involved, women's risk perceptions and barriers for participation in interventions.

Inclusion criteria for the participants were as follows: women with previous GDM participating in postpartum interventions with no restrictions on country, socio‐demographic factors (age, ethnicity, parity), socio‐economic factors or health‐related factors (comorbidity).

Intervention was defined as any pharmacological or non‐pharmacological initiative to prevent diabetes in women with previous GDM, provided and organized in any settings, and involving any stakeholders.

Qualitative or mixed reviews exploring women's risk perceptions and determinants for participating in preventive interventions or living a healthy lifestyle were eligible for inclusion. Exclusion criteria were as follows: overviews and reviews that incorporated theoretical studies or text and opinion as their primary source of evidence, and programmes that included women with established diabetes diagnosed before pregnancy.

### 2.3 Outcomes

The primary outcome was effectiveness in preventing diabetes presented with any estimates. Other outcomes were effect on lifestyle behaviour, data on the organization and stakeholders involved in the interventions. Furthermore, data on risk perceptions and participation barriers were extracted.

### 2.4 Data extraction

**Figure 1:**
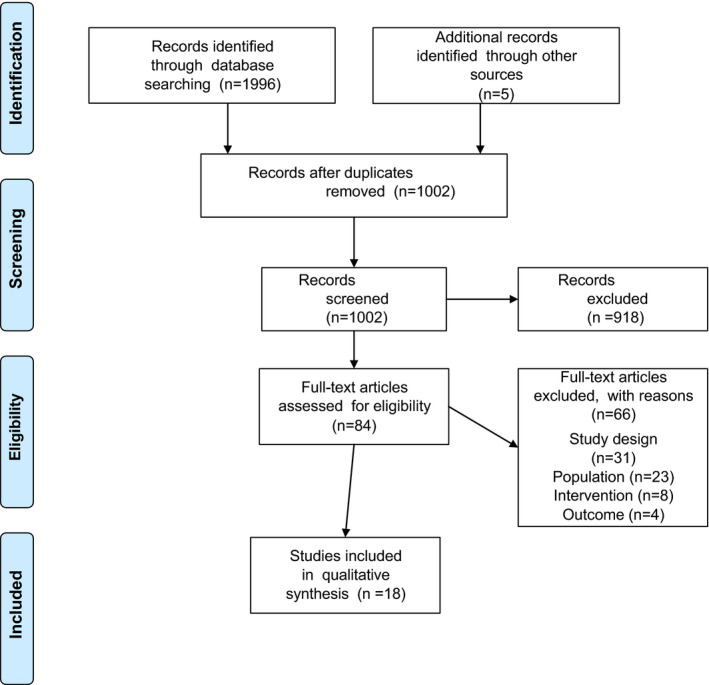
Flow diagram Note: Moher et al.^23^

Two reviewers independently screened the titles and abstracts of the identified eligible reviews. Secondly, the full text of all articles was screened by two reviewers when at least one reviewer deemed it potentially eligible. Any disagreement in assessment was solved by consensus. The selection process was recorded in a Preferred Reporting Items for Systematic Reviews and Meta‐Analyses (PRISMA) flow diagram (Figure 1).^23^ Data were extracted by one author and checked for accuracy by a second author using a structured initial data extraction form based on the research question. The form was piloted in four reviews to become familiar with the source results and to ascertain the ease of extraction of data within the reviewers. Any disagreement was solved by consensus. Characteristics of reviews and details of the interventions are presented in tables and analysed in a narrative summary.

### 2.5 Quality assessment

For reviews selected for retrieval, the reported quality assessment tool, rating and eventual use of reporting checklist (eg PRISMA checklist)^23^ were extracted. Quality assessment was conducted by couples of two reviewers using the standard JBI critical appraisal instrument for Systematic Reviews and Research Syntheses^21^ (Appendix 2). Reviews not meeting *a priori* number (a minimum of 5 ‘yes’) of 11 criteria were estimated to be of low methodological quality and were excluded. Any disagreements that arose between the reviewers were resolved by a third reviewer (AM).

## 3 RESULTS

The search identified 1996 articles, 999 of which were duplicates, and five additional records were identified from reference lists. Initial screening of abstracts and titles (DH, AM) left 84 articles for full‐text assessment for eligibility (DH, AM). After exclusion with reasons (Figure 1), 18 systematic reviews met the inclusion criteria. The total number of participants (women with previous GDM) ranged from *N* = 256^24^ to *N* = 122,877,^25^ and sample sizes of the primary studies included in the reviews ranged from 91 to 116,671.^25^ In total, 1,427,740 women were included in the overview. Eleven of the 18 systematic reviews consisted of quantitative primary studies, seven of which included RCTs,^18^, ^19^, ^26^, ^27^, ^28^, ^29^, ^30^ three included observational studies,^25^, ^31^, ^32^ and one included a mix of quantitative studies^24^ (Table 1). Six reviews consisted of mixed‐methods studies,^33^, ^34^, ^35^, ^36^, ^37^, ^38^ and one review included only qualitative studies.^39^

**Table 1:** Characteristics of included systematic reviews and details and characteristics of interventions

Study details	Systematic review	Participants	Aims	Description of interventions
Author year, context/setting country	Type of studies, number of studies included = n Meta‐analysis (MA), number of studies included in MA = mn Countries of origin of included studies Appraisal of studies, instrument used Reporting of review, checklist used Quality assessment, rating by Joanna Briggs Institute (JBI) checklist (0–11)	Total number = N		Details, characteristics of initiative Mode (of birth) Duration Follow‐up time (FU) Comparator(s) in randomized controlled trials (RCT), for example usual/standard care (UC)
Buelo et al., 2019 (34) Scottish Collaboration for Public Health Research and Policy, School of Health in Social Science, University of Edinburgh, United Kingdom (UK)	Quantitative (aim 1) Qualitative (aim 2) Mixed‐methods synthesis (aim 3) *n* = 28 RCT 12 Pre‐post studies 6 Interview 10 Australia 7 USA 5 Canada 4 China 1 Spain 1 Cochrane collaborations Risk of Bias Tool (RoB) Critical Appraisal Skills Programme (CASP) Preferred Reporting Items for Systematic Reviews and Meta‐Analyses (PRISMA) checklist JBI 10 1,2,3,4,5,6,7,8,10, 11	Women with previous gestational diabetes (GDM) *N* = 5,211	Explore Effectiveness of physical activity (PA) interventions to increase PA (reduce risk of diabetes (DM) Factors that women with previous GDM perceive influence their PA How these factors are addressed by the interventions	Lifestyle interventions or PA only (diet, PA, breastfeeding/child nutrition; diet, PA; diet, PA, mental health) Reporting of intervention components and study quality varied greatly Mode Group/individual Telephone, newsletters Websites, postcards, booklet Duration 12 weeks (w)–1 year (y) Follow‐up time (FU) 12 weeks (w)–1 year (y) Comparator(s) UC
Chasan‐Taber (27) Division of Biostatistics & Epidemiology, Dep. of Public Health, School of Public Health & Health Sciences, University of Massachusetts Amherst, MA, USA	Quantitative RCTs *n* = 9 5 pilot Australia 4 USA 3 China 1 Malaysia 1 No quality assessment JBI 6 1,2,3,8,10,11	Women with previous GDM *N* = 1,386	Provide researchers and practitioners with a comprehensive overview of RCTs of lifestyle interventions designed to reduce the risk of DM or DM risk factors among women with a history of GDM	Lifestyle interventions (diet, PA, breastfeeding) measured as impact on: T2DM incidence, weight, diet, PA, breastfeeding, and insulin resistance Weight (return to pre‐pregnancy weight if normal). 4/8 studies were conducted among women with current GDM or recent (within 2 m) Diet (healthy eating, low glycaemic index (GI), reduced calories, or <25/30% calories from fat) PA (moderate intensity) 150 min/w, 30 min/day for 3/5 times/w or 10,000 steps/day for 5 days/week Mode Group sessions/Individualized in‐person Telephone; Web‐based, text messaging, emails FU 10–12 months (m) Pilot studies 12 w ‐ 6 m Comparator Control arm or placebo
Dasqupta 2018 (32) Centre for Outcomes Research and Evaluation (CORE), Research Institute of the McGill University Health Centre, Montreal, Quebec, Canada	Quantitative Invited core outcome set (COS) review Qualitative synthesis *n* = 16 Australia 6 USA 6 China 2 Ireland 1 Spain 1 Malaysia 2 Canada 1 No quality assessment JBI 8 1,2,3,4,5,8,10,11	Women with previous GDM *N* = 5194 7 studies did not specify population	Gain insights on the factors that may enhance penetration and participation in Diabetes After Pregnancy prevention after GDM. Examine recruitment strategies and context	The Health Behaviour Change (HBC) after pregnancy interventions varied: 2 focused on PA only, incorporating pedometers 4 adapted the US Diabetes Prevention Program (DPP) 5 PA, healthy eating and breastfeeding 1 followed the Finnish‐DPS curriculum, emphasis on a low‐fat diet 1 adopted Mediterranean diet type of approach 1 compared low‐fat to a low‐GI diet 1 incorporated group cooking lessons Mode face‐to‐face, group lessons telephone contact Web‐based
Dennison 2019 (40) Primary Care Unit, Department of Public Health and Primary Care, Cambridge, UK	Qualitative studies *n* = 21 Australia 7 Tonga 1 Canada 3 Sweden 3 USA 5 UK 1 Ireland 1 Denmark 1 CASP all studies 8/10 JBI 10 (all but 9)	Women with previous GDM *N* = 903	Systematically synthesize the literature that focuses on the views of women with a history of GDM on reducing their risk of developing DM pp through lifestyle and behaviour changes.	Lifestyle and behaviour changes
Feng 2018 (26) Departments of Nutrition and Nursing, Sir Run Shaw Hospital, School of Medicine Zhejiang University, Hangzhou, China	Quantitative +MA *n* = 13 mn = 13 Prospective 6 Retrospective 7 USA 6, Ireland 1 Korea 2, Germany 1, Belgium 1, Australia 1, Italy1 New Castle‐Ottawa Scale PRISMA checklist JBI 10 1,2,3,4,5,6,7,8,9,11	Women with previous GDM *N* = 122,877 Sample size from 91–116,671	Investigate the association between lactation and development of type 2 DM in women with prior GDM.	Breastfeeding Duration 4–12 week FU 6 weeks−19 years Comparator No breastfeeding Breastfeeding <2–3 w
Gilinsky 2015 (27) School of Psychological & Health Sciences, UK	Quantitative +MA *n* = 13 mn = 11 RCTs 10 RCT cross‐over 1 Pre‐post 2 USA 5 Australia 5 China 1 Hong Kong 1 Malaysia 1 RoB 3/13 rated as low bias risk. PRISMA checklist JBI 8 1,2,3,4,5,6,8,9	Women with previous GDM *N* = 1,960	Review lifestyle interventions for women with prior GDM to report study characteristics, intervention design and study quality and explore changes in Diet, PA, and sedentary behaviour Anthropometric outcomes Glycaemic control and DM risk	Lifestyle PA and/or diet Breastfeeding Mode Face‐to‐face counselling Web‐based pedometer Telephone‐based education Group PA/education Electronic (SMS text/e‐mail) Newsletters Breastfeeding counselling 5‐day meal plan Free child care FU 6w−6y Comparator UC/no treatment Metformin and placebo Information on conventional dietary recommendations Written materials, two face‐to‐face education lessons (baseline, annually via phone/mail) Both groups advised to PA regularly (30 min, 3 times/w) Participants = own comparator
Goveia 2018 (29) Postgraduate Program in Epidemiology, Universidad Federal do Rio Grande do Sul, Porto Alegre, Brazil	Quantitative +MA *n* = 15 mn = 8 RCTs 15 USA 3, Australia 4, China 4, Spain 1, Malaysia 1, Israel 1, Ireland 1 RoB PRISMA checklist JBI 11	Women with previous GDM *N* = 2730	Compared lifestyle interventions: diet, PA or breastfeeding pp with UC without pharmacological treatment	Lifestyle interventions focused on changes in diet and PA. 3 only PA, 1 only on diet, 1 only on breastfeeding Mode 9 remote contact (phone, Internet, or postcards) 4 group sessions 11 individual face‐to‐face sessions (2 of these home visits, 9 held in the clinic). Duration Varied FU 3 m−7 years Comparator Standard/brief advice on diet and/or PA
Guo 2016 (30) School of Nursing, Central South University, Changsha, China.	Quantitative *n* = 12 6 pilot/feasibility RCTs 12 Australia 4 USA 3 China 4 Malaysia 1 The Cochrane RoB Methodological rigour of included studies PRISMA checklist JBI 10 1,2,3,4,5,6,7,8,10, 11	Women with previous GDM *N* = 2757 8: pp women with impaired glucose tolerance /impaired FBG or insufficient PA 10: pp women (4: 6 w pp 1: 2 y pp 4: 3 y pp 1: 4 y pp 2)	Systematically examine the components and effectiveness of pp lifestyle interventions in preventing T2DM in women with prior GDM Explore components of interventions that demonstrated a moderate effect on related measures of type 2 DM, insulin resistance, and weight.	Lifestyle interventions Mode PA (1 individual counselling +pedometer+ 5 telephone contacts +7 postcards) PA and psychosocial support (13 sessions, education, pedometer messaging, Internet forum) Diet (1 individual low‐GI diet education +2 handouts) (3 m sessions +dietary advice sheet reminders of PA) Diet and PA (8 individual meetings +2 tel. contacts) (6 home visits+3 tel. contacts) (7 individual sessions) Diet, PA, and psychosocial support (4 individual/tel. sessions) (self‐help booklet+10 tel. sessions) Diet, PA, and breastfeeding (6 tel.+ 2 individual+8 optional tel. sessions+3 tel. contacts) Diet, PA, and behaviour modification (16 individual meetings+3 group sessions) Duration 12 weeks−36 months (median 6 months). FU 3–69 months Comparator Basic advice, written lifestyle recommendations, infant safety, and general health Conventional healthy dietary recommendation. Standard dietary advice sheet and reminding of need for PA Health education materials Oral information about awareness of DM
Jones 2017 (18) Department of Nursing, College of Nursing and Health Sciences, University of Massachusetts Boston, USA	Quantitative studies RCTs *n* = 10 2 = protocols, no findings USA 5 Australian 4 Canada 1 The Cochrane Collaboration RoB JBI 8 1,2,3,4,5,6,10,11	Women with previous GDM *N* = 520 *N* = 3,140 incl. protocols	Synthesize current knowledge and practices around tailoring multimodal interventions for situational and cultural relevance to reduce type 2 DM risk in women with prior GDM.	Multimodal home‐based lifestyle modification intervention Theoretical framework/underpinning interventions 4/8 Social cognitive theory 2/8 Trans theoretical model 4/8 No specific theory reported; relevant constructs: self‐efficacy, risk perception, perceived benefits and barriers, health beliefs, self‐regulation, behavioural goals, social support, barriers to change Mode 7 Motivational interviewing or patient‐cantered counselling with experts 6 tel., 5 supplementary face‐to‐face sessions 4 mailings, 3 website, 2 supplementary text messages Duration Interventions are divided into 3 distinct phases: Prenatal (3‐trimester; 10‐14w) Early pp (6 weeks−6 months) Late pp (6–12 months) FU 3–12 months Comparator UC
Kaiser 2013 (35) Midwifery. University of Applied Sciences, Geneva, Switzerland	Mixed *n* = 18 Cross‐sectional surveys = 10 Cross‐sectional, and interviews = 8 Australia 8, Canada 2, USA 7, Sweden 1 No quality assessment JBI 9 1,2,3,4,5,6,7,10,11	Women with GDM *N* = 19847 Sample sizes 10–17,742	To describe the most significant findings of the studies that examined the prevalence and determinants of pp health behaviours (PA, dietary habits and/or weight loss) in patients with GDM	Identify factors that may impact the adherence to health behaviours specific for GDM patients What are the determinants of adherence or not to adequate pp health behaviours (PA, dietary habits, weight loss) and their potential determinants after GDM. Mode N/A Duration N/A FU 4 weeks−5 years
Middleton 2014 (31) Australian Research Centre for Health of Women and Babies, New Zealand	Cochrane review *n* = 1 RCT The Cochrane guideline GRADE JBI 10 1,2,3,4,5,6,7,9,10, 11	Women with a diagnosis of GDM in the index pregnancy. *N* = 256	To assess the effects of reminder systems to increase uptake of testing for T2DM or impaired glucose tolerance in women with a history of GDM.	Reminders of any modality (post, email, phone (direct call or short SMS text) to either women with a history of GDM or their health professional, or both. Mode 3 m pp postal reminders 1) to the woman only, 2) to the physician only, 3) to both. Women and physicians were contacted 3 times during 1 y FU Duration 1 y FU 1 y Comparator No reminder
Morton 2014 (25) Women's Health Research Unit London, UK	Mixed studies *n* = 11 RCTs 6 Observational 5 RoB JBI 9 1,2,3,4,5,6,7,10,11	Women with GDM. *N* = 10,968	Assess the effectiveness of various interventions that delay or arrest the progression from GDM to T2DM.	PA and/or dietary recommendation Breastfeeding Pharmacological interventions – Metformin Troglitazone 200 or 400 mg Pioglitazone 45 mg/day for 3 years Mode Advice by telephone, Individual counselling, lessons Recommendation Monitoring Food‐frequency questionnaire on DM by 14 years Questionnaires to calculate weekly energy expended in metabolic equivalent hours Duration FU 12 weeks−16 years Comparator No control/No intervention/ Placebo No breastfeeding (Ziegler)/ Women without GDM (Ratner) Routine advice on diet and PA plus low glycaemic dietary advice (Shyam) Alternate (Mediterranean) diet, approaches to hypertension (DASH) and healthy eating index (Tobias) Intensive advice diet, PA 3 m tel routine advice (Wein)
Nielsen 2014 (36) Department of International Health, Immunology and Microbiology, University of Copenhagen, Denmark	Mixed *n* = 58 RCTs, cohort, cross‐sectional, and qualitative studies Majority from high‐income countries: USA 28 Canada 8 Australia 10 New Zealand 1 Europe 7 No quality assessment JBI 5 1,2,5,10,11	Women with GDM *N* = 82,556 (1,053,345) *N* = 82,283 36 studies focusing on pp FU. (*N* = 273 15 studies focusing on GDM treatment) (*N* = 970,789 12 studies focusing on screening)	Investigate determinants and barriers to GDM care from initial screening and diagnosis to prenatal treatment and pp FU.	Screening during pregnancy Treatment of GDM during pregnancy and pp FU Healthy pp lifestyle interventions (diet or exercise)
Peacock 2014 (37) School of Nursing and Midwifery, Faculty of Health Sciences, The University of Queensland, Australia	Mixed quantitative/qualitative studies *n* = 30 RCTs 8 Observational 5 Cross‐sectional 8 Qualitative: Thematic Descriptive interpretive Modified grounded theory USA 11 Australia 11 Canada 2 Spain 1 Denmark 1 CONSORT (quality assessment of RCTs) JBI 9 1,2,3,4,5,6,8,10,11	Women previously diagnosed with GDM *N* = 184,502 Sample size: 10–177,420	Identify effective strategies and programmes to decrease the risk of T2DM in women who experience GDM, the barriers to participation, and the opportunities for midwives to assist women in prevention	Behavioural and pharmacological interventions intended to reduce maternal risk of T2DM
Pedersen 2017 (20) Public Health, Section for Health Promotion and Health Services, Aarhus University, Denmark	Quantitative +MA *n* = 10 RCTs = 9 Cluster RCT = 1, (44 medical centres, *N* = 2280) mn = 4 (951) Narrative synthesis Australia 4 USA 3 Asia 2 Europa 1 No quality assessment tool PRISMA checklist JBI 8 1,2,3,4,6,7,10,11	Women with a GDM diagnosis in the last pregnancy *N* = 3636	Review the evidence of effective behavioural interventions seeking to prevent T2DM	T2DM preventive health behaviours among women with previous GDM Behavioural interventions implemented within 2 years of the pp period PA +diet 5 trials measured effect on DM incidence FU 1y – approximately 4y
Tanase‐Nakao 2018 (33) Division of Maternal Medicine, Center for Maternal Foetal, Neonatal and Reproductive Medicine, Japan	Quantitative *n* = 9 Observational: 3 prospective cohort 4 cross‐sectional 2 retrospective cohorts (case control) USA 7 Germany 1 Korea 1 Data synthesis conducted by random‐effect MA Risk of Bias Assessment tool for non‐randomized studies (RoBANS) MOOSE guidelines JBI 9 1,2,3,4,5,6,7,8,9	Women with previous GDM *N* = 3,699	Review current findings on breastfeeding for type 2 DM prevention	Breastfeeding Duration FU 4 weeks – 5 years
Van den Heuvel 2018 (38) Division of Woman and Baby, University Medical Center Utrecht Netherlands	Mixed Narrative overview of the literature *n* = 71 No quality assessment JBI 6 1,2,3,7,10,11	Women prenatal, peri‐, and post‐ care	Provide a comprehensive and contemporary overview of the literature on eHealth in perinatal care and assess the applicability, advantages, limitations, and future of this new generation of pregnancy care	Electronic health (eHealth) including Web‐based informative programs, remote monitoring, tele‐consultation, and mobile device–supported care Mode eHealth, telemedicine
Van Ryswyk 2015 (39) Robinson Research Institute, The University of Adelaide, Australia	Mixed *n* = 42 Survey‐only 15 Interviews 18 Interviews and surveys 4 Interviews and focus groups 3 Focus groups 2 United States 12 Australia 10, Europe/UK 9 Canada 7 Brazil 2 Vietnam 1 Tonga 1 CASP PRISMA checklist JBI 9 1,2,3,4,5,6,7,10,11	Women with previous GDM *N* = 7,949	Identify factors that influence pp healthcare seeking for women who have experienced GDM through synthesis of results from qualitative and survey studies	

Abbreviations: BMI, body mass index; CASP, Critical Appraisal Skills Programme; COS, core outcome set; DM, diabetes mellitus; FBG, Fasting Blood Glucose; FU, follow‐up; GDM, gestational diabetes; GI, glycaemic index; m, month; JBI, Joanna Briggs Institute; MA, meta‐analysis; NS, not significant; OGTT, oral glucose tolerance test; PA, physical activity; pp, postpartum; PRISMA, Preferred Reporting Items for Systematic Reviews and Meta‐analyses; RoB, Cochrane collaborations Risk of Bias Tool; RoBANS, Risk of Bias assessment tool for non‐randomized studies; RCT, randomized controlled trial; UC, usual care; w, week; y, year.

The systematic reviews were conducted by researchers primarily in the Western world: Australia (*n* = 2), Canada (*n* = 1), Denmark (*n* = 2), the Netherlands (*n* = 1), New Zealand (*n* = 1), Switzerland (*n* = 1), the United Kingdom (*n* = 4) and the United States of America (*n* = 2), but one review was from researchers in Brazil, one was from Japan, and two were from China.

### 3.1 Quality assessment

The median rating was 8.6; thus, no review was excluded. In 12 reviews (18,25,26,28–31,33,34,37,39,40),^18^, ^38^, ^39^, ^40^ quality assessment of the primary studies was reported by use of either the Cochrane Collaboration Risk of Bias Tool, Critical Appraisal Skills Programme, New Castle‐Ottawa Scale^41^ or Grading of Recommendations, Assessment, Development and Evaluations (GRADE)^42^ (Table 1),^19^, ^37^ Guidelines for reporting were applied in nine of the reviews ^19^, ^25^, ^27^, ^28^, ^29^, ^30^, ^32^, ^33^, ^38^ by use of either the PRISMA checklist,^23^ the meta‐analysis of observational studies,^43^ strengthening the reporting of observational studies in epidemiology^44^ or the Cochrane guidelines.^45^

### 3.2 Interventions

Most reviews included lifestyle interventions (diet and physical activity), and six reviews also included interventions promoting breastfeeding.^24^, ^25^, ^26^, ^27^, ^28^, ^32^ Details are described in Table 1. Seven reviews included primarily diet and physical activity interventions,^18^, ^19^, ^24^, ^26^, ^27^, ^28^, ^29^ and one review focused on both effectiveness and determinants for adherence to physical activity.^33^ Six reviews included RCTs and cohort studies with both lifestyle and pharmacological interventions,^24^, ^33^, ^34^, ^35^, ^36^, ^37^, ^38^ The duration of the interventions varied substantially from 4 weeks^25^ up to 3 years.^18^, ^29^ The time to follow‐up also varied from 6 weeks to 16 and 19 years.^24^, ^25^ Duration of the screening programmes was not reported universally.^30^, ^35^, ^37^

Three reviews included postpartum screening interventions in women with previous GDM, for example reminders and determinants for participation.^30^, ^35^, ^46^

Ten of the quantitative reviews reported on measures of effectiveness, organization of interventions and the stakeholders involved.^18^, ^38^

Seven reviews including qualitative or mixed‐method studies described determinants or barriers for participation, adherence to changes in lifestyle and women's risk perceptions (Table 2).^31^, ^33^, ^34^, ^35^, ^36^, ^38^, ^39^

**Table 2:** Findings from systematic reviews including quantitative studies

Systematic review	Effectiveness of (breastfeeding, diet, physical activity, pharmacological) interventions, and screening on reducing diabetes	Stakeholders involved	Organisation
Buelo 2019 UK (34)	*Physical activity (PA)* 4/28 statistically significantly (SS) increased PA 14 had either mixed effectiveness or no changes in PA Reported intervention components and study quality varied greatly Interventions that incorporated childcare issues, social support and cultural sensitivities were associated with effectiveness	Healthcare professionals Doctors Practitioners Researchers	**‐**
Chasan‐Taber 2015 USA (27)	*Breastfeeding, diet, PA* 2/9 reported type 2 diabetes (T2DM) incidence Annual incidence rate 6.1% vs. 7.3% Incidence rate ratio (IRR) = 0.83, 95% confidence interval (CI) 0.47‐1.48 Breastfeeding vs. usual care (UC) 1/1 non‐significant (NS) difference Diet and exercise vs. placebo SS 53% risk reduction of T2DM incidence, *p* = 0.002 4/9 Diet vs. control, Low Glycaemic Index (GI) diet vs. UC SS improvements to on one or more dietary components 3 SS impact on weight change 4 NS impact on weight change 2 SS impact on Body Mass Index (BMI) change 1 NS impact on BMI Exercise vs. UC 3/9 SS impact on one or more measures of PA 4/9 Positive impact on biomarkers of insulin resistance (glucose measures) 2/4 NS	‐	‐
Feng 2018 China (26)	*Breastfeeding* 13 cohort studies included in the meta‐analysis (MA) 9/13 reported SS association with a lower T2DM risk Risk ratio (RR) 0.66, 95% CI 0.48‐0.90, I^2^ = 72.8%, *p* < 0.001) 3/13 Long‐term (>1–3 months (m) postpartum (pp) NS association with T2DM risk 1 USA study (RR 0.66, 95% CI 0.43‐0.99), SS regardless study design: prospective (RR 0.56, 95% CI 0.41–0.76); retrospective (RR 0.63, 95% CI 0.40–0.99), smaller sample size (RR 0.52, 95% CI 0.30–0.92, *p* = 0.024) Follow‐up (FU) >1 y (RR 0.75, 95% CI 0.56–1.00) (Adjusted RR 0.69, 95% CI 0.50–0.94)	‐	‐
Gilinsky 2015 UK (28)	Breastfeeding, diet, PA 3/13 reported on progression to T2DM (Ratner; Shek; Wein) Equally effective at reducing the rate of T2DM progression in women with previous gestational diabetes mellitus (pGDM) and without pGDM Numbers needed to treat higher among women with vs. women without previous GDM (pGDM) NS rate reduction in T2DM at 3 years (y) (Shek) and 51 m (Wein) Breastfeeding and sleep may offset T2DM risk after GDM MA found a SS 34% lower T2DM risk for any breastfeeding vs. no breastfeeding (Feng) Diet 6/11 favourable intervention effects PA 6/11 favourable intervention effects MA found SS weight loss was attributable to one Chinese population study (WMD = −1.06 kg (95% CI = −1.68−0.44) Lifestyle interventions NS change Fasting Blood Glucose (FBG) or T2DM risk Recruitment rates were poor but study retention good	Trained counsellor Exercise physiologist Dieticians Lifestyle behaviour case manager Research nutritionist Lactation consultant Peer educators (training and support from a multidisciplinary health professional team) Diabetes educators Research nurse	Hospital clinic and community health centre Hospital clinics
Goveia 2018 Brazil (29)	*Breastfeeding, diet, PA* MA found homogeneous (I^2^ = 10%), NS reduction of 25% T2DM incidence No beneficial changes in glycaemic levels (mean change from baseline of FBG, oral glucose tolerance test (OGTT) or haemoglobin A1c (HbA1c) Moderate reductions in weight (MD = −1.07 kg; −1.43−0.72 kg); BMI (MD = −0.94 kg/m^2^; −1.79 −0.09 kg/m^2^); and waist circumference (MD= −0.98 cm; −1.75 −0.21 cm) Only interventions soon after delivery (<6 months pp) were effective (RR =0.61; 95%CI: 0.40–0.94; p for subgroup comparison = 0.11) Effects were larger in studies with longer duration and FU Importance of maintaining support for lifestyle changes for a longer period, particularly given the women's frequently overwhelming tasks of motherhood	Lifestyle coach Nutrition coaching	Clinics Hospitals
Guo 2016 China (30)	*Diet, PA* Incidence of T2DM (FBG, or HbA1c). 5 lifestyle intervention vs. UC Annual mean T2DM incidence ranged from mean = 6.0% vs. mean = 9.3% NS, Effect size ranged from 0.05 – 0.40 among these 5 studies 7/10 evaluated FBG between the two groups 1 revealed a SS decreased FBG in the intervention group 5 effect size ranged from 0.004 to 0.50 2/10 evaluated HbA1c between group 1 SS decrease of HbA1c 7/10 reported at least a small effect size (> 0.20) on T2DM development 1 woman with GDM enrolled in Diabetes Prevention Program (DPP) had 12‐year interval (mean) on T2DM development (Ratner) Majority (75%) of studies only immediate or interim efficacy Increasing PA / Decreasing sedentary activity Pp weight gain/ Improving dietary outcomes Risk perception of T2DM	Trained counsellor Dietician Research nurse Exercise physiologist Case manager Diabetes educators Nutritionist Physicians healthcare professionals Trained interventionists	‐
Jones 2017 USA (18)	*Diet, PA* Diet, weight 7/8 SS reduced weight and hip and waist circumference, NS decreased weight, decreased dietary fat (Ferrara), Decreased weight 1 y FU (Nicklas) SS reduced total fat intake, total carb. intake and GI load (Reinhardt) NS decline in weight and insulin resistance; no changes in glucose levels (Kim), NS change in weight, BMI or insulin resistance (McIntyre) NS clinical improvement in eating behaviours, NS changes in glucose metabolism or body composition (Peacock) PA 8/8 No differences (Ferrara, Smith, Nicklas, Kim) NS % of women achieved goals, targets were not attained (Cheung) NS increased PA, majority failed to reach recommended PA levels (McIntyre) NS clinical improvement in PA (Peacock) NS changes in total level (Reinhardt)	Researchers Clinicians Communities Dieticians Lifestyle coach/ interventionist exercise physiologist	Home‐based settings
Middleton 2014 New Zealand (31)	Screening pp Postal reminders sent to, respectively: GDM women, GDM women and physicians, or physicians only Proportion of women having their first OGTT pp RR 3.87 (1.68–8.93) RR 4.23 (1.85–9.71) RR 3.61 (1.50–8.71) Proportion of women diagnosed with T2DM or showing impaired glucose tolerance or impaired FBG pp RR 1.57 (1.01–2.44) RR 1.78 (1.16–2.73) RR 1.69 (1.06–2.72) Low‐quality evidence for a marked increase in uptake of testing for T2DM Important to determine whether increased test uptake rates increase women's use of preventive strategies such as lifestyle modifications Other forms (email and telephone) reminders need to be assessed; more understanding of why some women fail to be screened pp is needed	Clinicians Health professional Physicians	Clinics University‐affiliated tertiary centre
Morton 2014 UK (25)	*Breastfeeding, diet, PA, pharmacological* Breastfeeding 15‐y risk of T2DM in women who breastfeed for >3 m vs. <3 m: 42% (95% CI 28.9–84.7) vs. 72% (60.5–84.7%) Protective effect on T2DM development remained SS after multivariate analysis (Hazard ratio (HR) 0.55, 95% CI 0.35–0.85, *p* < 0.001) SS decreased T2DM incidence after intensive lifestyle intervention with regular, individualized FU (3y): RR 0.50; *p* = 0.006 (Ratner) Diet and exercise RR 0.63 (95% CI 0.35–1.14) *p* = 0.12 (Wein) Diet and exercise RR 0.77 (95% CI 0.51–1.16) (Shek) Blood glucose 2 hour (h) post−75 g, load from baseline Low‐GI diet: Change in blood glucose, *p* = 0.025 (Shyam) Diet Effects of 3 dietary patterns (Tobias): 1‐unit interquartile range associated with 15% reduction, HR 0.84 (95% CI 0.73–0.96), The alternate Mediterranean diet, HR 0.86 (95% CI 0.73–1.03), *p* = 0.01; Dietary Approaches to Stop Hypertension), NS 17% reduction HR 0.77 (95% CI 0.64–0.93) alternate Healthy Eating Index Adjusted for BMI PA Comparing highest vs lowest quartiles of total PA over 16 y FU: SS 28% reduction in progression to T2DM (RR 0.72, 95% CI 0.55–0.96, *p* = 0.01) Women >7.5 metabolic equivalent hours/w vs. <7.5/w: SS 29% reduction in risk (RR 0.71, 95% CI 0.59–0.86, *p* < 0.001) (Bao) Pharmacological interventions Metformin SS 50% reduction in T2DM incidence >3y FU compared to UC (*p* = 0.006) HR 0.45 (95% CI 0.25–0.83) *p* = 0.009 (Buchanan), RR 0.47; *p* = 0.002 (Ratner) Troglitazone Prevention of Diabetes (TRIPOD) study (*n* = 266) on Hispanic 400 mg. SS reduction incidence, FU 30 m (HR 0.45, 95% CI 0.25–0.83, *p* = 0.009) Troglitazone (200 or 400 mg) in 42 Latino women, SS improvement in insulin sensitivity, FU 12 w 88 ± 22 (200 mg) 40 ± 22 (400 mg)/ 4 ± 14%, *p* = 0.03 (Berkowitz) SS decreased levels of fasting insulin concentrations, 20% ± 9% (400 mg) vs. +/−7% (200 mg) and 10% ± 10% (placebo), *p* = 0.03	Dieticians	‐
Peacock 2014 Australia (36)	*Diet, PA, pharma logical* Summary of identified studies Diabetes incidence rate SS decreased in the intervention group (5.4%) vs. placebo group (12.1%), *p* < 0.001 Diet NS returning to pre‐pregnant weight Intervention SS more effective in women without excessive gestational weight gain, *p* = 0.04 SS Weight reduction (95% CI: −7.6 to −0.5) and changes in dietary intake Reduction in weight in participants, *p* = 0.03 Eating patterns were changed during the index GDM pregnancy (protein *p* = 0.01, fibre *p* = 0.002) but not sustained pp PA SS leisure time PA increased in first year in women post GDM (*p* = 0.002) NS differences in PA and weight loss NS average time of PA (mean 60 (0–540) min/week) increased NS 10,000 steps on 5 or more days not reached Pharmacological Lifestyle changes (58% {48–66, 95%CI}) and Metformin (31% {17–43, 95%CI}) reduced the incidence of diabetes Lifestyle intervention (*p* = 0.002) and Metformin (*p* = 0.006) reduced the risk of T2DM compared to placebo and control Results supported a class effect of Thiazolidinedione drugs to enhance insulin sensitivity, reduce insulin secretory demands and preserve pancreatic b‐cell function in intervention group, *p* = 0.01 Group sessions demonstrated a potential to improve perceptions of healthiness in women but NS		
Pedersen 2017 Denmark (20)	*Diet, PA* No specific intervention or components were found superior NS reduction of T2DM incidence (tendency only) SS pooled estimate of absolute risk reduction (−5.02 per 100 (95% CI: −9.24;−0.80) SS effect in the subgroup of participants >40 y (T2DM incidence 8% in intervention group vs. 20% in control group, *n* = 175, *p* = 0.018 Tendency of poorer effect starting during pregnancy or very early pp (≤6 w) vs. interventions started >6 w pp SS changes were found for PA but not for diet Biomarkers of insulin resistance Generally, results were consistent within trials 2 showed NS effect on fasting glucose in spite of a SS intervention effect on other measures of insulin resistance	Trained dieticians Exercise physiologist Trained research nurse	Medical centres Fitness centres
Tanase‐Nakao 2018 Japan (33)	*Breastfeeding* 6/9 reported results in favour of breastfeeding regards to T2DM incidence, 3/9 reported null results 2–4 w pp breastfeeding tends to lower the risk of T2DM compared with women with shorter period. SS effect with FU>2 y FU<2 y = OR 0.77, (95% CI 0.01–55.86) 2–5 y = OR 0.56, (95% CI 0.35–0.89) >5 y = OR 0.22, (95% CI 0.13–0.36) Exclusively breastfeeding for 6–9 weeks pp lower the risk compared with women giving formula feeding (OR 0.42, 95% CI 0.22–0.81)	‐	‐
Van der Heuvel 2018 Netherlands (38)	Screening pp eHealth in GDM care has evolved most notably of all perinatal appliances of eHealth the last 3 years (smartphone‐facilitated remote blood glucose monitoring, management of medication schedules through Web‐based or SMS‐facilitated feedback systems, and telephone review service to support and supervise glycaemic control) Decrease in planned and unplanned visits by 50% to 66%, whereas no unfavourable differences in glycaemic control, maternal, and neonatal outcomes occurred Advantages of eHealth implementation in perinatal care: Patient satisfaction and engagement, fewer clinic visits, clinician satisfaction, remote monitoring, access to care in low‐ and middle‐income countries Disadvantages and indistinct impacts: reimbursement, legal issues, technical issues, limited A‐level evidence, health outcome and costs pp screening after GDM with telephone FU (RCT) (Roozbahani) SS reduced FBG levels in mothers with GDM and increased the rate of pp screening test	Obstetricians	Outpatients clinics Hospitals Tertiary hospital

Abbreviations: BMI, body mass index; CI, confidence interval; DPP, Diabetes Prevention Program; FBG, Fasting Blood Glucose; FU, follow‐up; m, month; GI, Glycaemic Index; h, hour; HbA1c, haemoglobin A1c; HR, Hazard ratio; IRR, incidence rate ratio; MA, meta‐analysis; MD, mean difference; NS, not significant; OGTT, oral glucose tolerance test; PA, physical activity; pGDM, previous gestational diabetes mellitus; pp, postpartum; p, p‐values*; RR, risk ratio; SS, statistically significant; T2DM, type 2 diabetes; UC, usual care; w, week; y, year.

* *p*‐values and authors of primary studies only if reported in the systematic review.

### 3.3 Intervention effect

Twelve reviews presented data on incidence or risk reduction of postpartum diabetes.^18^, ^32^, ^33^, ^37^ All but two also presented estimates on risk associations between behaviours and postpartum diabetes.^29^, ^37^ However, meta‐analyses were not performed due to heterogeneity of the study populations. Thus, the effectiveness of the interventions was presented in both descriptive and analytic terms (Table 2. The five reviews that included interventions promoting breastfeeding found a positive impact; one review concluded that exclusively breastfeeding for 6–9 weeks significantly reduced the risk of diabetes compared with formula at more than 2 years of follow‐up.^32^ A meta‐analysis demonstrated reduction of the incidence of diabetes by 34%,^25^ and breastfeeding for at least 12 weeks reduced the risk of diabetes significantly at 15 years of follow‐up.^25^

Two reviews on pharmacological interventions presented 31% and 50% decrease in diabetes after treatment postnatally with metformin and troglitazone, respectively, after 3 years of follow‐up.^36^

The majority of lifestyle interventions reported at least a small effect on diabetes development. Peacock et al. reported on both diet and physical activity interventions and showed a reduction in incidence of diabetes compared to control and placebo by 58%.^36^ A review including RCTs with diet and exercise interventions showed a reduction in incidence of diabetes by 53%.^26^ Components included were intake of reduced calories and regular physical activity at moderate intensity (150 min per week) for 6 months.^26^ In another review of any form of lifestyle, interventions in five RTCs found a significantly decreased incidence of diabetes in the subgroup of women above 40 years, with a follow‐up of 1–4 years after birth.^19^

A mixed‐method review found in four of 28 RCTs an increased level of physical activity 3–12 months after intervention; social support, childcare issues and cultural background impacted significantly on the effectiveness of interventions.^33^ However, only a third of the RCTs on exercise showed an effect on measures of physical activity and on biomarkers of insulin resistance.^26^ Peacock et al. examined various lifestyle and pharmacological interventions (individualized exercise plan, motivational interviewing programme, dietary components) and found that only dietary interventions reduced weight and changed dietary intake, although they were more effective in women without excessive gestational weight gain.^36^ Pedersen et al. concluded that lifestyle interventions in RCTs increased physical activity but not changes in diet (20).^19^ Another review concluded that behavioural interventions had a significant effect on eating patterns during pregnancy and leisure time physical activity in the first year postpartum.^36^

Timing, duration and recruitment to interventions Timing was of importance regarding effectiveness; early postpartum (2–6 months) interventions were most effective.^28^ Jones et al. showed the start‐up time for interventions was divided into three distinct periods: prenatal and early and late postpartum.^18^ Pedersen et al. found that lifestyle interventions started during pregnancy were less effective than interventions implemented 6 weeks postpartum.^19^ Furthermore, effect was superior if interventions lasted more than 1 year, but effect was less at 3 years of follow‐up than after 1 year.^19^

Recruitment method impacted upon participation rates. A review including eHealth interventions with a RCT design concluded that postpartum screening with follow‐up by telephone increased screening rate and reduced fasting blood glucose levels in women with previous GDM.^37^ Recruitment during pregnancy or the early postpartum period increased the participation rate more than 40%, especially if face‐to‐face contact was used in the GDM care setting.^31^ In contrast, a mailed invitation and/or telephone contact later in the postpartum period decreased the participation to less than 15%.^31^

### 3.4 Perceived risk and barriers

Mixed‐method studies found that the effectiveness of the interventions depended on incorporation of factors of importance for participation.^33^ These were typically interacting behavioural factors, for example lack of support from family and professionals, cultural sensitivities and lack of resources and information. Typical barriers for women's participation were lack of information during pregnancy, lack of knowledge of risk factors, preventive behaviours and, explicitly, the role of physical activity.^34^, ^35^, ^36^, ^38^, ^39^ For some women, the importance of physical activity was perceived to be relevant only to control blood glucose and lose weight during pregnancy. Thus, only 7% of women believed that physical activity would decrease the risk of diabetes later in life.^36^ One review that included physical activity interventions found that use of pedometers was not effective.^33^ Another review concluded that although women may continue eating healthy postpartum, some stopped being concerned with what they ate because they perceived that their diet no longer had an impact on the health of the child.^35^ Furthermore, during breastfeeding some women increased their food intake.^35^ The review found that only a minority of women were conscious of their high risk of developing diabetes later in life.^35^ Despite an intention to maintain a healthy lifestyle, most women did not, and only one in three reported a sufficient level of daily physical activity.^35^

### 3.5 Organization of interventions

Twelve of the systematic reviews reported on organizational aspects of the interventions (Tables 2 and 3).^18^, ^37^, ^38^, ^39^ ranging from unspecified to involving several settings: participants’ home, community‐based practice or health centre, GDM care setting, public/urban hospital, university health system, private practice, women's hospital, clinic or ward, pregnancy service, urban antenatal clinic, private obstetrician clinic, GDM clinic or unit, medical centre at tertiary hospital and university prenatal clinic.^31^ One review reported behavioural interventions in home‐based settings only,^18^ and another review reported interventions in fitness centres.^19^ Women expressed preferences for programmes that allowed access from home (eg Internet‐based or telephone intervention), thereby overcoming accessibility issues.^36^ Women also expressed a need for support from a lifestyle coach and provision of family friendly programmes.^36^

**Table 3:** Findings from systematic reviews including qualitative studies

Systematic review	Determinants and barriers for diabetes prevention (lifestyle behaviours, diet, physical activity, and screening)	Stakeholders involved	Organisation
Buelo 2019 UK (34)	Determinants Putting others before yourself, putting off lifestyle change, lack of support from healthcare professionals, being a healthy role model for families, accounting for childcare issues, social support and cultural sensitivities Interventions (Random control trials (RCTs) that incorporated these factors were associated with effectiveness Education about how to reduce future risk of type 2 diabetes mellitus (T2DM) and pedometers in interventions were not associated with effectiveness	Healthcare professionals Doctors Healthcare providers Practitioners Researchers	**‐**
Dasqupta 2018 Canada (32)	Participation (calculated as the proportion of) those invited who actually enrol in different intervention programs varied substantially Penetration (coverage of the target population) calculated as the proportion invited to participate in interventions for preventing diabetes was 85–100% When recruitment occurred during pregnancy or early postpartum (pp), participation was >40% or more, especially if face‐to‐face contact was used within the gestational diabetes mellitus (GDM) care setting, but participation <15% in mid/late pp with mailed invitation and/or telephone contact	Lactation consultant Dietician Health coach Nurses Physical activity (PA) specialists Physicians Exercise physiologist	Participants’ home Community‐based practice Community health centre GDM units, care settings Public/urban hospitals University health system Private practices Women's wards Pregnancy service Urban antenatal clinics Private obstetrician clinic Medical centre University prenatal clinics
Dennison 2009 UK (40)	Lifestyle change influences Determinants (interacting influences on pp behaviour): Role as mother and priorities; social support from family and friends; demands of life; personal preferences and experiences; diabetes risk perception and information; finances and resources; format of interventions Barriers Women identified themselves primarily as mothers who prioritized their family above themselves, and needed resources, time, energy, information and support to encourage healthy diets and levels of activity Important to adapt interventions to the target population and facilitate family‐friendly changes because the mother's own diabetes risk was unlikely to motivate change without her perceiving benefits for her children Some of the most beneficial aspects of groups (e.g. forming supportive relationships) are impractical for most to commit to in the long term	Physicians Clinic staff Obstetric and healthcare providers Professionals Supportive relationships Dieticians Case manager Nurse	Hospital‐based specialist clinic GDM clinic Diabetes obstetric service Hospital‐affiliated academic clinics General practices Multidisciplinary team
Kaiser 2013 Switzerland (35)	Adherence to health behaviours: Health behaviours, impact on adoption of: women's own perception of health, risk perception, risk and knowledge regarding diabetes, impact of health beliefs and psychosocial factors, social support, self‐efficacy Determinants Information during pregnancy, recall of advice/remembered receiving diabetes prevention information, perception and awareness of risk of diabetes, knowledge of risk factors and preventive behaviours, knowledge on diabetes and role of PA, social support from partner, family and friends, appropriate childcare Partner/family support, high social support, high self‐efficacy, companions, community safety, transportation, centre‐based programme Barriers Lack of assistance for child care/ constraints related to children, lack of time, time constraints, enjoyment of activity, necessity to prevent later health problems, self‐perceived health status, continuing support and education post partum, beliefs about health and illness, perceived risk, self‐efficacy, perceived personal control, beliefs in the benefits and barriers of lifestyle modification, financial constraints, lack of motivation/fatigue, difficulty at work, mental distress, role perceptions, cultural expectations, psychological wellbeing, psychosocial constructs, body mass index (BMI) Barriers to PA Lack of assistance with childcare, time constraints, physical complaints, lack of knowledge, lack of safety, family responsibilities, partner and family attitudes and beliefs, social isolation Characteristics: BMI, age, education, employment, marital status, living with children, ethnicity	Healthcare providers Midwives Nurses Multidisciplinary care teams Health educator Nutrition education therapist Husband/partner Family and friends Partner/family	Maternity care units
Nielsen Denmark (36)	Determinants for healthy lifestyle pp (diet, PA) Despite women expressed they intended to live a healthy lifestyle pp, it was generally not achieved. Among women with GDM in the past 6 months (m) −2 year (y) unhealthy diet was prevalent, only 34% reported sufficient PA. Women with previous GDM do not perceive themselves to be at increased risk of future diabetes. 90% of women (US population) recognized GDM as a risk factor for future diabetes, only 16% believed they themselves were at high risk, though the proportion increased to 39% when asked to estimate their risk assuming they maintained their current lifestyle. 40% of women with a history of GDM were very worried about developing diabetes in the future, 46% a little worried and 14% not worried at all. Some women increase their food intake during breastfeeding Determinants for diet Self‐efficacy was associated with high vegetable consumption, ability to cook healthy foods, and reporting that healthy diet is not a difficult change and that dislike of healthy foods by other household members is not a barrier for them. Moreover, self‐efficacy when busy and not reporting a dislike of healthy foods by others at home were associated with high fruit consumption Determinants for PA Independently associated with high self‐efficacy and social support. *Barriers for PA* Lack of time and/or energy, child care support, motivation, knowledge about GDM, social support, support from health care provider, enjoyment of PA, not feeling well, emotional distress, financial barriers, domestic responsibilities such as cooking, feeling of solitude, dullness and isolation from family and friends, poor body image, bad weather, considering oneself to be too young to be on a restricted diet, obstacles at work, unsuitable local neighbourhood, no access to exercise equipment, cultural expectations, bad weather; considering oneself to be too young to be on a restricted diet; unsuitable local+neighbourhood or no access to exercise equipment; cultural expectations e.g. needs of women come last in the family. Women who perceived themselves to be at no or slight risk of diabetes were less likely to modify their lifestyle. Many women tried to continue eating healthy pp to protect their health However, some pp women felt they no longer had to worry about what they were eating as it would no longer impact the health of the baby. Intentions of healthy lifestyle may be there, but many do not succeed in continuing modifications. May be influenced by their perception of risk of future diabetes and particularly by self‐efficacy and social support Barriers to screening pp Not considering the test necessary, declining testing, unable to complete test, testing not affordable, uninformed, lack of understanding of need for test, practice being too busy, time pressure, lost requisition, recent delivery experience, baby's health issues, adjustment to the new baby (emotional stress, feeling overwhelmed and lack of time and burden of child care), concerns about pp and future health (feeling healthy and not in need for care, and fear of receiving bad news), experiences with medical care and services (dissatisfaction with care and logistics of accessing care).	Health care providers Obstetricians Gynaecologists Primary care providers Family practice physicians Maternal‐foetal specialists Family physicians Endocrinologists Internists	Health care system Health care centres Hospital settings High‐risk pregnancy settings. Antenatal care clinics Private hospital Non‐private clinic Public hospitals Gyn/obs‐specialist practice setting General practitioners Obstetricians´ private practices
Peacock 2014 Australia (37)	*Barriers to lifestyle change* Some interventions are effective, but lifestyle changes are difficult to translate into everyday life. Women with previous GDM (pGDM) need to overcome barriers and be supported in making the behavioural changes necessary A woman's ability to follow a healthy lifestyle depends on her psychological wellbeing, as well as social and cultural support The difficulty balancing household expectations and leading a healthy lifestyle and the complexities of women's motivations Health behaviours ‘the feeling of abandonment’ by health care providers and the hospital pp in contrast to the intensive monitoring during their pregnancy, recognition that lifestyle changes are difficult Participation determinants Preference for a programme of support that allowed access from home (e.g. internet based) and/or support from ‘lifestyle coach’. Early pp interventions using telephone experienced a greater percentage of weight loss and lifestyle behaviour changes. Increased social support and facilitating increased PA self‐efficacy, as well as a ‘‘family friendly’’ approach, may help increase lifestyle recommendations A healthy diet (more vegetables and less fried foods) was too great a change from their current behaviours PA Concern about progression to diabetes were not observed to increase their levels of PA or lose weight as advised during pregnancies. Women exercised in pregnancy to control their blood glucose levels, whereas pp exercise was perceived as important only to assist weight loss. Only 7% of women believed that PA pp would decrease their risk, despite the education provided during pregnancy A proportion of women were not ready (reported ‘‘preaction’’ phase) for both undertaking sufficient levels of PA and taking steps to lose weight. Many reported ‘readiness to change’ behaviour; however, the majority remained overweight *Barriers to PA* Negatively influence, initiation/engagement in PA, lack of assistance with child care, insufficient time, financial constraints, fatigue, work issues, lack of social support	Primary carers Midwives Specialist midwives Endocrinologists Obstetricians Diabetes educators Dieticians Multi‐disciplinary team members General Practitioners	Midwife‐led GDM care clinics Multidisciplinary team care clinics
Van Ryswyk 2015 Australia (39)	While women were often knowledgeable about risk and prevention of T2DM. They faced multiple barriers to undertaking preventive behaviours. A need for support of lifestyle changes and more pro‐active postpartum care was identified. Determinants for seeking healthcare pp: Knowledge and perception of risk of diabetes, knowledge of complications of diabetes (for mothers and/or offspring), and knowledge of preventing future diabetes. Attitudes towards pp FU of GDM, pp oral glucose tolerance test (OGTT), reminders for FU or fasting blood glucose (FGB).	Clinicians Health professionals Family Clinical staff Healthcare providers	Postpartum clinics

Abbreviations: BMI, body mass index; FBG, Fasting Blood Glucose; FU, follow‐up; GDM, gestational diabetes; m, month; MA, meta‐analysis; NS, non significant; p, p‐values (if p‐values are reported), previous GDM (pGDM); PA, physical activity; pp, postpartum; OGTT, oral glucose tolerance test; RCT, randomized controlled trial; SS, statistically significantly; w, week; y, years, T2DM, Type 2 diabetes mellitus; y, year.

### 3.6 Stakeholders involved

Fifteen of the systematic reviews reported on specific stakeholders involved in the interventions (Tables 2 and 3).^18^, ^37^, ^38^, ^39^ These ranged from a few unspecified healthcare professionals and researchers up to nine different professions (trained counsellor, exercise physiologist, dietician, lifestyle behaviour case manager, research nutritionist, lactation consultant, peer educator, diabetes educator and research nurse).^27^ The most prevalent stakeholders involved were dieticians,however, their role was not described in detail. One review concluded that midwives played an important role as primary carers, as they were ideally positioned to educate and engage women in lifestyle programmes during pregnancy and following the postpartum period.^36^ In postpartum screening programmes that only involved obstetricians.^37^ both women and clinicians were more satisfied with eHealth programmes with remote monitoring, and planned and unplanned clinic visits were reduced by 50% and 66%, respectively.^37^ In contrast to the intensive GDM monitoring during pregnancy, the women reported they felt abandoned by healthcare providers postpartum and found difficulties balancing household demands and following a healthy lifestyle.^36^ A need was identified for more proactive support and postpartum care, together with the need for information regarding the risk and complications of diabetes for themselves and their offspring.^38^ Furthermore, interventions may benefit from forming support groups or relationships, although these may impractical to be committed to in the long term.^39^

## 4 DISCUSSION

### 4.1 Principal findings

This overview included 18 systematic reviews, seven of which consisted of qualitative or mixed studies. Eleven of the reviews reported on effectiveness, organization as well as stakeholders involved in the interventions.

### 4.2 Effectiveness, timing, duration and recruitment to interventions

The scope, components and duration of the interventions varied. A majority of the lifestyle programmes including breastfeeding, physical activity, healthy diet and pharmacological interventions were effective in reducing the incidence of diabetes or delaying its onset in this high‐risk population. Effectiveness of the interventions depended on timing, duration and incorporation of factors of importance for participation, such as professional and social support. The findings are in accordance with earlier reports of intensive lifestyle and metformin interventions (the Diabetes Prevention Program, DPP); both interventions were highly efficient in reducing progression to Type 2 diabetes,^15^ after 10 year, the incidence was reduced by 35%–40%.^47^

Despite the long term increased risk among the women, the interventions were in general followed up for only a few months, but showed the importance to maintain support for lifestyle changes for a longer period.^40^ The most comprehensive meta‐analysis found that among women (average age 30 years) minimal changes in anthropometric measures over a short period translate into a 25% risk reduction of diabetes.^40^ A need for long‐term follow‐up was similarly underlined by qualitative systematic reviews, where a need was expressed for GDM follow‐up and proactive support from lifestyle coaches and healthcare professionals.

Participation in screening and lifestyle programmes is promoted by early postpartum recruitment, especially with face‐to‐face contact in GDM healthcare setting. Thus, implementation up to 6 weeks postpartum proved to be superior; preventive interventions should be long‐lasting to be most effective although some interventions showed a decreased effect after 3 years.

### 4.3 Organization of interventions and stakeholders involved

Most interventions involved professionals from different fields; the roles or skills of the professionals have to be described in detail to legitimate their involvement. Midwives play an important role as primary carers for women during pregnancy and childbirth and may play a part after birth in engaging women in lifestyle programmes,^36^ trusted dieticians and coaches for training in family friendly programmes are needed. Furthermore, the findings stressed the importance of acknowledgment of the women's need of support from professionals and families for participation. This is in accordance with a previous review, which also found it was necessary to involve the healthcare system as well as the family context in preventive programmes.^48^

### 4.4 Perceived risks and barriers for participation

The findings revealed lack of knowledge of risk factors among the women. Furthermore, the qualitative reviews found that women reported a lack of postpartum care and demonstrated a lack of knowledge that GDM begets type 2 diabetes.^6^ Thus, the women expressed a need for reliable information and support from professionals (eg information about risk of diabetes for the woman as well as their child).

A theory‐based study found effectiveness of a health promotion intervention among adults at high risk of diabetes.^49^ However, only a few reviews reported on this issue. Jones et al. reported that only 50% of the primary studies specified a theoretical framework used for the intervention, for example the social cognitive theory and the transtheoretical model.^18^

### 4.5 Strengths and weaknesses of the study

An overview of reviews considers the highest level of evidence; the methodology followed was rigorous and comprehensive, with duplicate screeners throughout title and abstract screening, full‐text review, quality assessment and data extraction.

This overview explored effectiveness and determinants for preventive interventions among women following GDM. According to the aim, drawing a broader range of evidence, this overview added value as it included qualitative data in order to explore typical barriers for participation in certain interventions.^38^ Our findings are complemented by Dennison et al.’s recommendations for prevention programmes, for example to inform from trusted sources (dieticians or healthcare providers) about the risks and wider benefits of healthier lifestyle and to advocate for adequate exercise.^39^

Only three reviews were from emerging countries, thus limiting the validity of our findings and conclusions to settings in the Western world.

A review on clinicians’ views was not included due to criteria for participants; the findings showed gaps in postpartum screening practice, and a need to improve collaboration among stakeholders and education about GDM.^46^

### 4.6 Future preventive interventions

The need for interventions after GDM is evident, and most findings and recommendations are reproducible for programmes in a local healthcare setting. Supporting an intended healthy lifestyle postpartum seems to be the challenge; unhealthy diet and insufficient level of daily physical activity were very common among the women.

Participation barriers regarding screening should be taken into account, for example healthcare provider (specialist or family physician) not seeing the patient, lack of communication and collaboration between healthcare providers and the women, inconsistent guidelines or lack of familiarity with guidelines, and no awareness about the woman's history of GDM.^35^

To motivate women to take advantage of healthcare opportunities, automatic reminders in patient charts or electronic medical records would be beneficial. Postpartum screening programmes may increase participation by using telephone follow‐up and eHealth. Behavioural interventions are often provided face‐to‐face; however, eHealth may prove less costly; a meta‐analysis found strong evidence for use of mobile phone apps for lifestyle modification in diabetes.^50^ The lack of awareness among the women for the need of screening should be addressed, and healthcare providers should adhere to newest available guidelines.

A combination of approaches may be most appropriate, for example online information, target‐setting and options to arrange video calls with dieticians and contact with local groups of women who also had experienced GDM.^39^ eHealth interventions had a multilevel field of application and advantages for patients and clinicians in screening programmes.^37^

Programmes may include promotion of screening, breastfeeding, focus on adequate physical activity, healthy diet and eventually pharmacological treatment. Focus should be on education and provision of knowledge on advantages of breastfeeding and life‐long healthy lifestyle and support from professionals. Similarly, families should be supported,^33^ as the combination of increased patient empowerment and pregnancy care could lead to greater satisfaction and efficiency.^37^

Motivational factors are of importance; thus, early initiation of long‐lasting programmes should be preferred.^19^ Interventions may profit from forming support groups or relationships as well as involving families, for example regarding child care, as women find it difficult to balance the expectations of their new role. On the other hand, advantages of eHealth implementation, for example remote monitoring in screening, may be obvious (patient satisfaction, engagement, fewer clinic visits).

Preventive interventions and research reporting long‐time follow‐up are urgently needed. Furthermore, knowledge is needed on which lifestyle components and pharmacological treatments are most effective in specific subgroups. Research on methods to empower the women to adapt a healthy lifestyle is needed.

## CONFLICT OF INTEREST

The authors declare no conflicts of interests with respect to the research, authorship or funding of this research project.

## AUTHOR CONTRIBUTIONS

All authors have given final approval of the version to be published. Anne‐Mette Hedeager Momsen contributed substantially to design, acquisition, analysis and interpretation of data and drafting the manuscript. Diana Høtoft participated in screening and data extracting, quality assessment and presentation of the included reviews in tables. Diana Høtoft also contributed to revision of the manuscript. Lisbeth Ørtenblad contributed to the design, revised and evaluated the manuscript, approved the final version. Vibeke Lynggaard and Helle Terkildsen Maindal made contributions to design, analysis and interpretation of data, revising it critically for important intellectual content. Finn Friis Lauszus evaluated included publications and revised the manuscript. Rubab Hassan Agha Krogh and Jens Juel Christiansen evaluated included publications and revised the manuscript. Claus Vinther Nielsen contributed substantially to design, acquisition, analysis and interpretation of data and the drafting of the manuscript.

## Data Availability

Data are available in the included systematic reviews and Tables 1‐3.

## Appendix 1

### Search Strategy

**Table:**

Database	Interface	Date	Hits
PubMed	PubMed	March 2019	402
EMBASE	Elsevier	March 2019	535
CINAHL	Ebsco	March 2019	92
Web of Science Core Collection	Clarivate	March 2019	64
The Cochrane Library	Wiley	March 2019	23
Joanna Briggs Institute EBP Database	OVID	March 2019	40
			in total 1156 hits

**Table:**

Inclusion/Exclusion criteria	Years: 2009 1st January – 29th March 2019 Languages: English, Danish, Norwegian, Swedish Publications: Systematic Review, Review, Meta‐analysis

Notes.

- Search terms and inclusion/exclusion criteria adjusted to each database.
- Doublets are if possible sorted by RefWorks.
- Search strategy for each database presented on the following pages.

PubMed d. 07/03/19.

((((((systematic[sb] OR meta‐analysis[pt] OR meta‐analysis as topic[mh] OR meta‐analysis[mh] OR meta analy*[tw] OR metanaly*[tw] OR metaanaly*[tw] OR met analy*[tw] OR integrative research[tiab] OR "review"[ti] OR integrative review*[tiab] OR integrative overview*[tiab] OR research integration*[tiab] OR research overview*[tiab] OR collaborative review*[tiab] OR collaborative overview*[tiab] OR systematic review*[tiab] OR comparative efficacy[tiab] OR comparative effectiveness[tiab] OR outcomes research[tiab] OR indirect comparison*[tiab] OR ((indirect treatment[tiab] OR mixed‐treatment[tiab]) AND comparison*[tiab]) OR Embase*[tiab] OR Cinahl*[tiab] OR systematic overview*[tiab] OR methodological overview*[tiab] OR methodologic overview*[tiab] OR methodological review*[tiab] OR methodologic review*[tiab] OR quantitative review*[tiab] OR quantitative overview*[tiab] OR quantitative synthes*[tiab] OR pooled analy*[tiab] OR Cochrane[tiab] OR Medline[tiab] OR Pubmed[tiab] OR Medlars[tiab] OR handsearch*[tiab] OR hand search*[tiab] OR meta‐regression*[tiab] OR metaregression*[tiab] OR data synthes*[tiab] OR data extraction[tiab] OR data abstraction*[tiab]) AND (("2009/01/01"[PDat]: "3000/12/31"[PDat]) AND (Danish[lang] OR English[lang] OR Norwegian[lang] OR Swedish[lang])))) AND ((((((((((((((((((((("Maternal Health"[Mesh]) OR "Maternal Behavior"[Mesh]) OR "Life Style"[Mesh]) OR "Healthy Lifestyle"[Mesh]) OR "Health Behavior"[Mesh]) OR "Risk Reduction Behavior"[Mesh]) OR "Self Care"[Mesh]) OR "Self‐Management"[Mesh]) OR "Patient Education as Topic"[Mesh]) OR "Health Education"[Mesh]) OR "Health Promotion"[Mesh]) OR "Motivation"[Mesh]) OR "Weight Reduction Programs"[Mesh]) OR "Exercise Therapy"[Mesh]) OR "Exercise"[Mesh]) OR "Distance Counseling"[Mesh])) OR ((("diabetes risk" OR "cardiometabolic risk" OR "physical activity" OR "diabetes prevention" OR "Prevention programme" OR "Cooperative behavior" OR "Support" OR "Diabetes self‐management" OR "Self‐management support" OR "Tailored care" OR "Empowerment" OR "Counseling" OR "Educational intervention" OR "Dietary intervention")) OR ("coping" OR "behavior change" OR "e‐counseling")))) AND ((((("health services"[MeSH Terms] OR "maternal health services"[MeSH Terms] OR Maternal welfare OR "postnatal care"[MeSH Terms] OR "intersectoral collaboration"[MeSH Terms] OR "delivery of health care, integrated"[MeSH Terms] OR "Health care team" OR "Patient care team" OR "Integrated care" OR "Multi‐sectoral partnership" OR "Community" OR "Community‐based" OR "Municipality" OR "Real‐life setting" OR "Real‐world environment" OR "outpatient services" OR Collaboration OR Intercollaboration OR "Interprofessional collaboration"))) OR "Delivery of Health Care"[Mesh]) OR "Ambulatory Care"[Mesh])) AND ("diabetes, gestational"[MeSH Terms] OR "gestational diabetes" OR "postpartum diabetes" OR "diabetes in pregnancy" OR "postpartum period"))) AND (("2009/01/01"[PDat]: "3000/12/31"[PDat]) AND (Danish[lang] OR English[lang] OR Norwegian[lang] OR Swedish[lang])))) OR (((((((((((((((((((((("Maternal Health"[Mesh]) OR "Maternal Behavior"[Mesh]) OR "Life Style"[Mesh]) OR "Healthy Lifestyle"[Mesh]) OR "Health Behavior"[Mesh]) OR "Risk Reduction Behavior"[Mesh]) OR "Self Care"[Mesh]) OR "Self‐Management"[Mesh]) OR "Patient Education as Topic"[Mesh]) OR "Health Education"[Mesh]) OR "Health Promotion"[Mesh]) OR "Motivation"[Mesh]) OR "Weight Reduction Programs"[Mesh]) OR "Exercise Therapy"[Mesh]) OR "Exercise"[Mesh]) OR "Distance Counseling"[Mesh])) OR ((("diabetes risk" OR "cardiometabolic risk" OR "physical activity" OR "diabetes prevention" OR "Prevention programme" OR "Cooperative behavior" OR "Support" OR "Diabetes self‐management" OR "Self‐management support" OR "Tailored care" OR "Empowerment" OR "Counseling" OR "Educational intervention" OR "Dietary intervention")) OR ("coping" OR "behavior change" OR "e‐counseling")))) AND ((((("health services"[MeSH Terms] OR "maternal health services"[MeSH Terms] OR Maternal welfare OR "postnatal care"[MeSH Terms] OR "intersectoral collaboration"[MeSH Terms] OR "delivery of health care, integrated"[MeSH Terms] OR "Health care team" OR "Patient care team" OR "Integrated care" OR "Multi‐sectoral partnership" OR "Community" OR "Community‐based" OR "Municipality" OR "Real‐life setting" OR "Real‐world environment" OR "outpatient services" OR Collaboration OR Intercollaboration OR "Interprofessional collaboration"))) OR "Delivery of Health Care"[Mesh]) OR "Ambulatory Care"[Mesh])) AND ("diabetes, gestational"[MeSH Terms] OR "gestational diabetes" OR "postpartum diabetes" OR "diabetes in pregnancy" OR "postpartum period")) AND ((Meta‐Analysis[ptyp] OR Review[ptyp] OR systematic[sb]) AND ("2009/01/01"[PDat]: "3000/12/31"[PDat]) AND (Danish[lang] OR English[lang] OR Norwegian[lang] OR Swedish[lang]))) Filters: Publication date from 2009/01/01; Danish; English; Norwegian; Swedish.

402 references.

Embase.com 07/03/2019.

535 references.

Cinahl 07/03/2019.

**Table:**

Terms	Search Options	Actions	
S31	S29 AND S30	**Limiters** ‐ Published Date: 20090101–20191231 **Search modes** ‐ Boolean/Phrase Language: Danish, English, Norwegian, Swedish	**View Results** (92) **View Details**
S30	(MH "Meta Analysis") OR TI meta analys* OR AB meta analys* OR TI Metaanaly* OR AB metaanalys* OR (MH "Literature Review+") OR TI systematic review* OR AB systematic review* OR TI systematic overview* OR AB systematic overview* NOT (PT commentary OR PT letter OR PT editorial OR MH animals+)	**Search modes** ‐ Boolean/Phrase	**View Results** (144,029) **View Details**
S29	S3 AND S19 AND S28	**Search modes** ‐ Boolean/Phrase	**View Results** (2,209)
S28	S20 OR S21 OR S22 OR S23 OR S24 OR S25 OR S26 OR S27	**Search modes** ‐ Boolean/Phrase	**View Results** (1,190,888)
S27	TX "Health care team" OR "Patient care team" OR "Integrated care" OR "Multi‐sectoral partnership" OR "Community" OR "Community‐based Municipality" OR "Real‐life setting" OR "Real‐world environment" OR "outpatient services" OR "Collaboration Interprofessional" OR "collaboration Intercollaboration"	**Search modes** ‐ Boolean/Phrase	**View Results** (242,983) **View Details**
S26	(MH "Ambulatory Care")	**Search modes** ‐ Boolean/Phrase	**View Results** (10,339)
S25	(MH "Health Care Delivery, Integrated")	**Search modes** ‐ Boolean/Phrase	**View Results** (9,250)
S24	(MH "Health Care Delivery+")	**Search modes** ‐ Boolean/Phrase	**View Results** (283,186)
S23	(MH "Postnatal Care+")	**Search modes** ‐ Boolean/Phrase	**View Results** (4,450)
S22	(MH "Maternal Welfare")	**Search modes** ‐ Boolean/Phrase	**View Results** (1,013)
S21	(MH "Maternal Health Services+")	**Search modes** ‐ Boolean/Phrase	**View Results** (25,063)
S20	(MH "Health Services+")	**Search modes** ‐ Boolean/Phrase	**View Results** (871,492)
S19	S4 OR S5 OR S6 OR S7 OR S8 OR S9 OR S10 OR S11 OR S12 OR S13 OR S14 OR S15 OR S16 OR S17 OR S18	**Search modes** ‐ Boolean/Phrase	**View Results** (952,956)
S18	TX "Physical activity" OR "Diabetes risk" OR "Cardiometabolic risk" OR "Diabetes prevention" OR "Prevention programme" OR "Cooperative behavior" OR "Support" OR "Diabetes self‐management" OR "Self‐management support" OR "Tailored care" OR "Empowerment" OR "Counseling" OR "Educational intervention" OR "Dietary intervention"	**Search modes** ‐ Boolean/Phrase	**View Results** (481,298) **View Details**
S17	(MH "Exercise+")	**Search modes** ‐ Boolean/Phrase	**View Results** (96,626)
S16	(MH "Therapeutic Exercise+")	**Search modes** ‐ Boolean/Phrase	**View Results** (47,463)
S15	(MH "Weight Reduction Programs")	**Search modes** ‐ Boolean/Phrase	**View Results** (2,262)
S14	(MH "Coping+")	**Search modes** ‐ Boolean/Phrase	**View Results** (30,827)
S13	(MH "Motivation+")	**Search modes** ‐ Boolean/Phrase	**View Results** (79,309)
S12	(MH "Health Promotion+")	**Search modes** ‐ Boolean/Phrase	**View Results** (57,143)
S11	(MH "Health Education+")	**Search modes** ‐ Boolean/Phrase	**View Results** (110,079)
S10	(MH "Patient Education+")	**Search modes** ‐ Boolean/Phrase	**View Results** (68,616)
S9	(MH "Self Care+") OR (MH "Self‐management")	**Search modes** ‐ Boolean/Phrase	**View Results** (42,712)
S8	(MH "Behavioral Changes") OR (MH "Risk taking behavior")	**Search modes** ‐ Boolean/Phrase	**View Results** (26,391)
S7	(MH "Health Behavior+")	**Search modes** ‐ Boolean/Phrase	**View Results** (86,112)
S6	(MH "Life Style+") OR (MH "Life Style Changes")	**Search modes** ‐ Boolean/Phrase	**View Results** (188,090)
S5	(MH "Maternal Behavior") OR (MH "Maternal‐child health")	**Search modes** ‐ Boolean/Phrase	**View Results** (5,673)
S4	(MH "Primary Health Care") OR (MH "Secondary Health Care") OR (MH "Tertiary Health Care")	**Search modes** ‐ Boolean/Phrase	**View Results** (56,516)
S3	S1 OR S2	**Search modes** ‐ Boolean/Phrase	**View Results** (14,890)
S2	TX "Gestational diabetes" OR "Postpartum diabetes" OR "Diabetes in pregnancy" OR "Postpartum period"	**Search modes** ‐ Boolean/Phrase	**View Results** (13,545) **View Details**
S1	(MH "Diabetes Mellitus, Gestational+")	**Search modes** ‐ Boolean/Phrase	**View Results** (5,560)

92 references.

Web of Science 07/03/2019.

64 references.

The Cochrane Library 07/03/2019 11:37:16.

Comment:

**Table:**

ID	Search	Hits
#1	MeSH descriptor: [Diabetes, Gestational] explode all trees	743
#2	#1 with Cochrane Library publication date Between Jan 2009 and Jan 2019, in Cochrane Reviews	23

23 references.

Database(s): Joanna Briggs Institute EBP Database ‐ Current to February 27, 2019 Search Strategy:

**Table:**

No.	Searches	Results
1	gestational diabetes.mp. [mp=text, heading word, subject area node, title]	48
2	Postpartum diabetes.mp. [mp=text, heading word, subject area node, title]	2
3	Diabetes in pregnancy.mp. [mp=text, heading word, subject area node, title]	18
4	Postpartum period.mp. [mp=text, heading word, subject area node, title]	44
5	1 or 2 or 3 or 4	89
6	limit 5 to ((evidence summaries or systematic reviews) and yr="2009 ‐Current")	40

40 references.
